# How Far Do the Complaints of Patients with Parkinson's Disease Reflect Motor Fluctuation? Quantitative Analysis Using a Portable Gait Rhythmogram

**DOI:** 10.5402/2012/372030

**Published:** 2012-12-13

**Authors:** Hiroya Utsumi, Hiroo Terashi, Yohei Ishimura, Tomoko Takazawa, Yasuyuki Okuma, Mitsuru Yoneyama, Hiroshi Mitoma

**Affiliations:** ^1^Department of Neurology, Tokyo Medical University, Tokyo 160-0023, Japan; ^2^Department of Neurology, Shizuoka Hospital, Juntendo University, Izunokuni-shi 410-2295, Japan; ^3^Mitsubishi Chemical Group Science and Technology Research Center, Inc., Yokohama-shi 227-8502, Japan; ^4^Department of Medical Education, Tokyo Medical University, Tokyo 160-0023, Japan

## Abstract

In advanced-stage Parkinson's disease (PD), motor fluctuation is a frequent and disabling problem. Assessment of motor fluctuation depends on patient's subjective self-statement. We examined whether the subjective fluctuation matched the objective motor fluctuation defined by gait disorders. Using a new device, the portable gait rhythmogram, we recorded gait cadence and acceleration continuously over the 24-hour period in 54 patients with PD and 17 normal controls, for the quantitative evaluation of motor fluctuation. The patients were asked to estimate motor fluctuation every hour. In 44 of 54 patients, changes in the cadence were associated with simultaneous changes in acceleration. We examined the subjective fluctuation in these 44 patients who were confirmed to have motor fluctuation. Nineteen (82.7%) of 23 patients who felt no fluctuation showed distinct gait disorders. During off time, they walked with marked short or bradykinetic stepping. No matching changes were observed in either the cadence or acceleration in 11 (52.4%) of 21 patients who perceived motor fluctuation. No synchronization was noted in 30 (68.2%) of the 44 patients, between the times of subjectively assessed motor fluctuation and those of quantitative analysis of gait disorder. This discrepancy suggests that the objective continuous recording of the cadence and acceleration is necessary to understand motor fluctuation.

## 1. Introduction

Dopamine-replacement therapy at the early stage of Parkinson's disease (PD) improves motor and nonmotor complications. In the advanced stage, however, motor fluctuation is a frequent and disabling problem. Up to 50% of patients exhibit motor fluctuation and resistance to medication after the first five years of treatment [1–3]. Motor fluctuation is estimated by explanations or patients' diaries, though they are subjective and not quantitative [4, 5]. How far are the complaints of patients reliable, that is, how far do they reflect the actual motor fluctuation? To address this question, motor fluctuation must be objectively quantified for comparison.

Gait disorders are cardinal symptoms in PD and can be easily quantified since gait movements are defined by three parameters: the cadence (steps/min), the floor reaction force, and stride [6, 7]. Thus, gait disorders can be physiologically a good model for the quantitative analysis of difficulties in motor execution. To examine motor fluctuation, we developed a long-term monitoring portable device, gait rhythmogram (PGR) that measures distinguishably the accelerations induced by gaits [8–11]. The PGR can record continuously the walking pattern of patients during daily life activities. Our previous studies using this new device showed that the range of changes in cadence and gait acceleration was narrow, suggesting that PD patients find it difficult to shift gait parameters in response to varying situations during walking [10, 11]. Furthermore, we reported a decrease in cadence during the off time, when the PD patients manifested bradykinesia or marked instability, and an increase in the cadence while the patients walked with short-stepping, festination, or freezing of gait [8, 9]. The results suggested that various complex pathophysiological changes underlying parkinsonian symptoms can be simply expressed as changes in cadence. Long-term monitoring of changes in gait cadence, therefore, can theoretically be used to provide a quantitative measure of motor fluctuation. Since this method appears to detect deficits in motor execution, it can, therefore, detect motor fluctuation with a higher sensitivity than the examination of movement poverty by monitoring acceleration by various movements [12].

The aim of the present study was to determine the relationship between complaints of PD patients and true motor fluctuation. For this purpose, daily profiles of motor symptoms must be identified. In the present study, we first improved the analysis method used for PGR. In addition to measuring the cadence, we simultaneously measured changes in the amplitude of gait accelerations, since such amplitude correlates with floor reaction forces and the floor reaction forces are known to decrease during off time [8, 9]. By simultaneously tracing alterations in cadence and acceleration, a better identification of motor fluctuation would be possible. We also compared the changes in these gait parameters with those by subjective fluctuation. Subjective fluctuation was assessed based on diaries written by patients.

## 2. Methods

### 2.1. Subjects

Using this PGR, we recorded continuously the daily profiles of 54 patients with PD (age: 71.4 ± 7.0 years, mean ± SD, 30 men and 24 women). They represented all patients admitted to Tokyo Medical University Hospital between June 2009 and May 2010, who could walk unaided and showed no peak-dose dyskinesia during “on” time. They included 4 patients with modified Hoehn and Yahr stage 1.5, 14 with stage 2, 10 with stage 2.5, 24 with stage 3, 1 with stage 3.5, and 1 with stage 4. The clinical status was examined using Unified Parkinson's Disease Rating Scale (UPDRS) motor score “on” state [13] (Table 1). We also included 17 height-matched normal control subjects (age: 64.7 ± 4.5 years, 8 men and 9 women). Matching for age and height was based on the finding that gait cycle and floor reaction forces are influenced by these two parameters [9]. Informed consent was obtained from all subjects. All procedures were conducted in accordance with the guidelines of the Ethics Committee of our institution.

### 2.2. Measurements

#### 2.2.1. Monitoring Gait Accelerations

The PGR is a small device (size, 8 × 6 × 2 cm, weight, 80 g) that measures three dimensionally (*a*_*x*_, *a*_*y*_, and *a*_*z*_) the accelerations accompanied by (1) limb and trunk movements and (2) those induced by step-in and kick-off during gait, as reported previously [8, 9]. The PGR is attached to the waist of the patient and records the above signals at a sampling rate of 10 msec. The data are automatically stored in a micro SD card. When recording is completed, the absolute values of acceleration vectors (*a*; *a* ^2^ = *a*_*x*_^2^ + *a*_*y*_^2^ + *a*_*z*_^2^) are calculated and graphically displayed on the PC. A fully charged PGR can achieve 40 consecutive hours of recording.

#### 2.2.2. Identification of Acceleration Induced by Gait Motion

The acceleration vectors caused by stepping can be distinguished from those by other limb and trunk movements or by unexpected artifacts, based on the mathematical method of “pattern matching” [8, 9]. Since the acceleration wave induced by gait motion was morphologically similar in each patient, correlation was mathematically tested between the concerned waves and the template. After the identification of gait-induced acceleration, the peak-to-peak interval is automatically detected to calculate the duration of a single gait cycle, in addition to the amplitude of gait accelerations.

#### 2.2.3. Long-Term Monitoring of the Gait Cycle and Acceleration

Changes in gait cadence (steps per minute) and gait acceleration cycle were examined during 24 hrs. Cadences and accelerations represent the mean values recorded every hour. Data were excluded if the number of steps was less than 20 per hour.

### 2.3. Criteria for Identification of Gait Off and Gait Good

We classified the patients into two groups, the “*probable gait off*” group and “*probable gait good*” group, based on changes in the cadence throughout the day. The validity of the classification was then tested by the changes in acceleration. When corresponding changes were observed between the two gait parameters, we classified these patients into “*definite gait off*” group and “*definite gait good*” group. In patients of the latter two groups, we examined the relationship between the gait findings and subjective complaints. In contrast, if no corresponding change was observed, we reserved the assessment of the gait profile and excluded these patients for comparison between gait and subjective records.

#### 2.3.1. Identification of Gait Off

Gait fluctuation was suspected when patients walked with a large fluctuation of the cadence, that is, deviation from the intersubject mean ± 1SD of the normal controls. The interindividual mean ± SD of the normal controls was 110 ± 12 steps/min. Thus, the cutoff levels set in the present study were <98 and >122 steps/min. These candidates were tested by the following two procedures. First, all activity-related changes were excluded. A simultaneous increase in cadence and acceleration was interpreted to be caused by high activities. Second, gait off was considered to occur only when the cadence change was accompanied with a decrease in acceleration under the interindividual mean − 2SD of the normal control. Since the interindividual mean ± SD of the normal control was 2.78 ± 0.42/sec^2^, the cut-off level was set <1.94 m/sec^2^.

#### 2.3.2. Identification of Constantly Gait Good

Patients were considered to walk well throughout the day without motor fluctuation when walking was achieved with a small fluctuation that was within the interindividual mean ± 1SD of the normal control. Since the interindividual mean ± SD of the normal control was 110 ± 12 steps/min, the cut-off levels were set between 98 and 122 steps/min. These candidates were then tested by changes in the acceleration. Absence of gait fluctuation was considered when the CV (coefficient variance) of the acceleration was less than the interindividual mean + 1SD of the control. We considered that the unusual fluctuation occurred when the acceleration CV was beyond mean + 1SD of the control. Since the interindividual mean ± SD of the normal controls was 0.20 ± 0.04, the cut-off level was set <0.24.

### 2.4. Assessment of Subjective Fluctuation

Patients were asked to classify their symptoms into *good*, *not good,* and *bad*. At every hour, patients recorded in their diaries these subjective comments.

### 2.5. Statistical Analysis

Statistical analysis was performed using the Student's *t*-test. A *P* value of <0.05 denoted the presence of a significant difference.

## 3. Results

### 3.1. Cadence and Acceleration Fluctuation in 24-hours Recordings

#### 3.1.1. Patients with Gait Off

**Figure 1:**
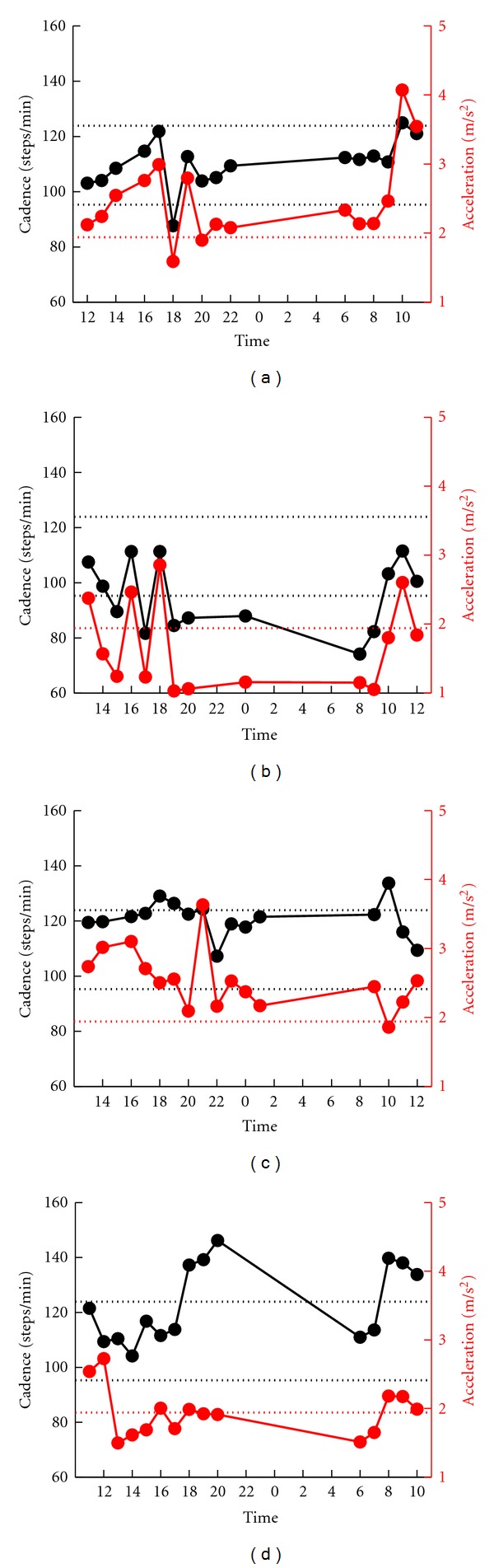
Serial changes in gait cadence and acceleration during daily activities. Examples of patients who showed fluctuation in the cadence. Left ordinate: the cadence (black continuous line and symbols), right ordinate: acceleration (red continuous line and symbols), abscissa: time, black dotted line: mean cadence ± 1SD of normal subjects, and red dotted line: mean acceleration − 2SD of normal subjects. The patients in (a), (b), and (c) showed synchronized fluctuations in cadence and acceleration, whereas patient in (d) showed desynchronization.

Forty-one of the 54 patients showed a large cadence fluctuation that deviated from the interindividual mean ± 1SD of the normal controls (patients with *probable gait off*). First, we excluded the activity-related changes from these cadence fluctuations. For example, in the patient shown in Figure 1(a), both the cadence and acceleration increased at 1000 and 1100, when she was doing housekeeping work and walking outside according to patient's diary. After excluding the activity-related changes, we checked whether or not the cadence change was accompanied by a decrease in gait acceleration, that is, below the interindividual mean − 2SD of the normal control.

Two types of fluctuation were observed. The first type was observed in patients who showed bradykinetic stepping. The decreased cadence corresponded to the decrease in the amplitude of the acceleration (Figures 1(a) and 1(b)). Patient no. 38 shown in Figure 1(a) walked slowly at 1800. The bradykinetic decrease in cadence was associated with a simultaneous decrease in acceleration at 1800. In Patient no. 31 shown in Figure 1(b), the cadence slowed at 1500, 1700, 1900, 2000, 2400, 800, and 900, and the acceleration decreased throughout these times. Thus, simultaneous changes were confirmed at 1500, 1700, 1900, 2000, 2400, 800, and 900. Another type of fluctuation was observed in patients with the marked short stepping or freezing of gait. The acceleration decreased in amplitude, which corresponded with the increase in cadence. Patient no. 5 (Figure 1(c)) walked with marked short-stepping at 1400, 1500, 1700, and 600. At 600, the patient appeared to walk with short stepping or freezing and the acceleration simultaneously decreased in amplitude. However, the increase in cadence at 1700 was an activity-related change since acceleration increased at the same time.

Of the 41 patients with *probable gait off*, 37 patients were assessed as *definite gait off* based on the simultaneous changes in cadence and acceleration. No simultaneous changes were observed in the two parameters in the other four patients (Figure 1(d)).

#### 3.1.2. Patients Showing Gait Good

**Figure 2:**
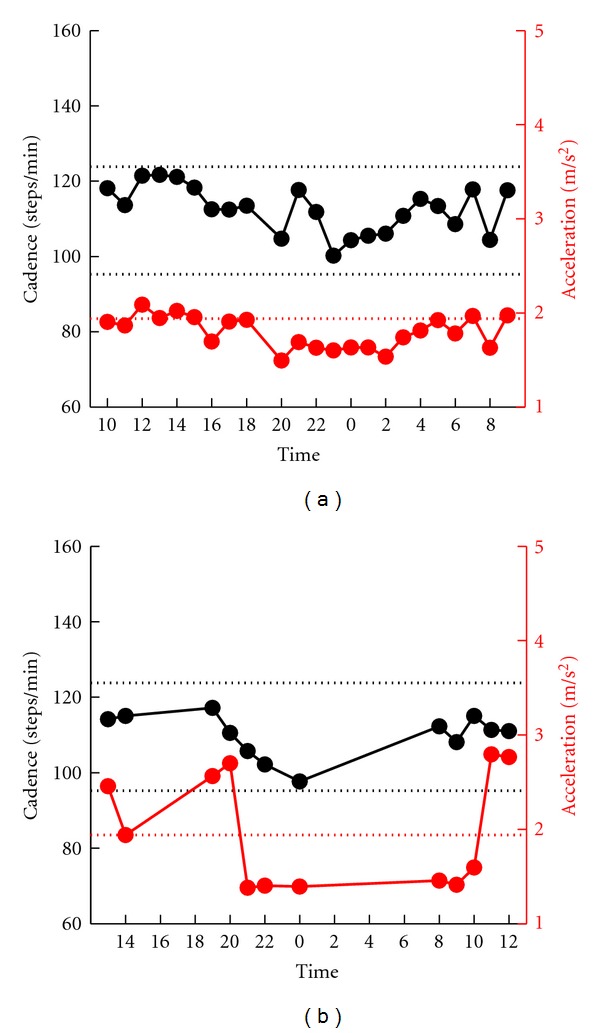
Serial changes in gait cadence and acceleration during daily activities. Examples of patients who showed no fluctuation in the cadence. Left ordinate: the cadence (black continuous line and symbols), right ordinate: acceleration (red continuous line and symbols), abscissa: time, black dotted line: mean cadence ± 1SD of normal subjects, and red dotted line: mean acceleration − 2SD of normal subjects. Fluctuations in acceleration were small in the patient shown in (a), and large in the patient shown in (b).

Thirteen patients of the 54 patients showed small cadence fluctuation that was within the interindividual mean ± 1SD of the normal controls (patients with *probable gait good*). Examination of these patients showed CV of acceleration below the mean + 1SD (0.24) in 7 patients. For example, Patient no. 2 (Figure 2(a)) with CV = 0.13 showed small fluctuation also in acceleration. These patients were classified into the* definite gait good* group. The CV of the other 6 patients was more than the mean + 1SD (0.24).  Figure 2(b) shows an example of a patient with CV = 0.27 in whom the acceleration showed larger fluctuations.

### 3.2. Comparison between Subjective Fluctuation and Gait Fluctuation

Finally, we examined the subjective complaints of the 37 patients with *definite gait off* and 7 patients with *definite gait good*. The results of these patients are shown in Table 2.

#### 3.2.1. Patients Who Did Not Notice “Off”

**Figure 3:**
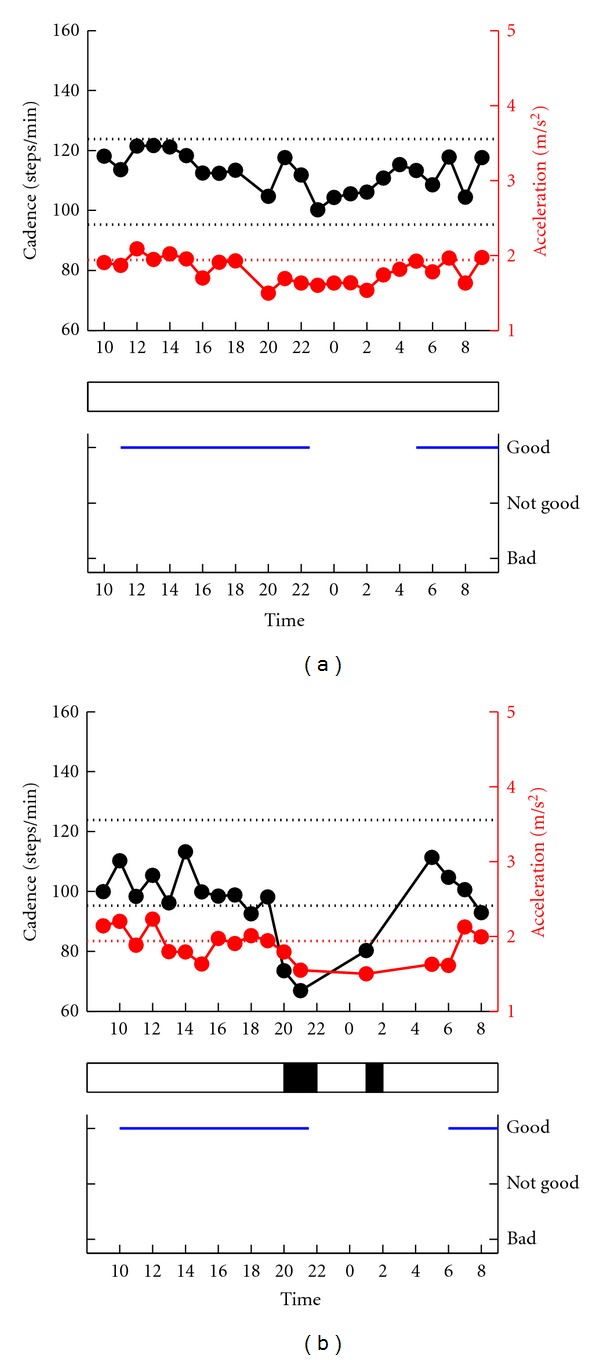
Comparison between gait fluctuation (*top*) and subjective fluctuation (*bottom*). Examples of patients who subjectively felt no motor fluctuation. Top: serial changes in gait cadence and acceleration during daily activities. Left ordinate: the cadence (black continuous line and symbols), right ordinate: acceleration (red continuous line and symbols), abscissa: time, black dotted line: mean cadence ± 1SD of normal subjects, red dotted line: mean acceleration − 2SD of normal subjects. The determined gait off is indicated by black rectangle below the top figure. The lack of awareness of motor fluctuation was in agreement with the lack of recorded changes in gait fluctuation in (a), where the two sets of data were in disagreement in (b).

Of the 44 patients, 23 patients did not notice any motor fluctuation. Four patients (17.3%) reported no changes in the cadence and acceleration (Patients no. 1–4). The results of Patient no. 2 are shown in Figure 3(a). However, the PGR showed a distinct fluctuation in gait rhythm in the other 19 (82.7%) patients (Patients no. 5–23). For example, patient no. 15 shown in Figure 3(b) walked a with slow gait cycle and decreased acceleration at 2000, 2100, and 100 due to a marked bradykinesia. During the objective gait off time, the patient did not experience the worsening of motor symptoms including gait. The time during which the patients were not aware of gait fluctuation varied,and included the morning, early afternoon, evening and night (see patients no. 5–23, Table 2). No significant difference was observed in UPDRS-III between the patients with synchronization and those lacking such synchronization.

#### 3.2.2. Patients Who Noticed “Off”

**Figure 4:**
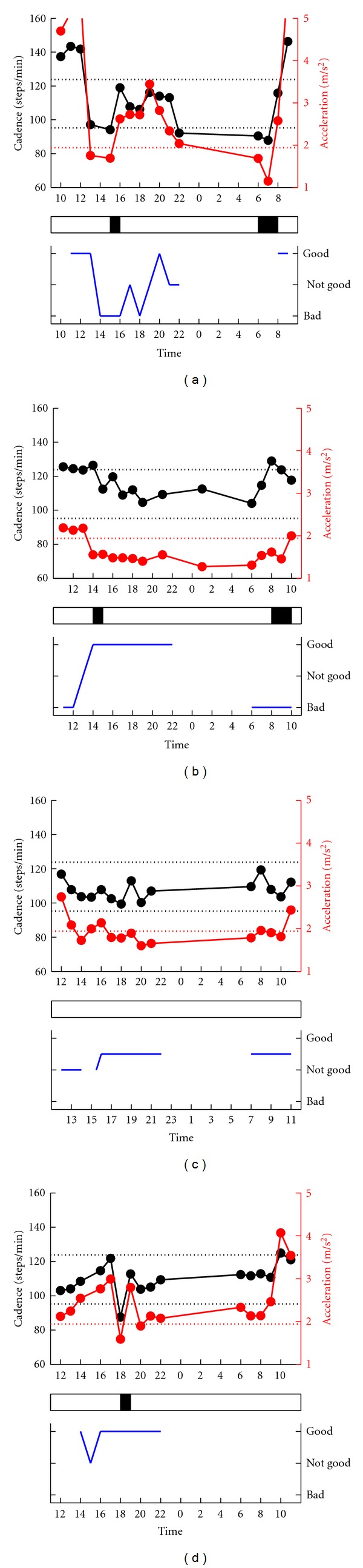
Comparison between gait fluctuation (*top*) and subjective fluctuation (*bottom*). Examples of patients who reported motor fluctuations. Top: serial changes in gait cadence and acceleration during daily activities. Left ordinate: the cadence (black continuous line and symbols), right ordinate: acceleration (red continuous line and symbols), abscissa: time, black dotted line: mean cadence ± 1SD of normal subjects, red dotted line: mean acceleration − 2SD of normal subjects. The determined gait off is indicated by black rectangle below the top figure. Awareness of motor fluctuation was in agreement with the recorded changes in gait fluctuation in (a) and (b), but not in (c) and (d).

The other 21 (48%) patients reported motor fluctuation. In 10 of these 21 patients (47.6%), there was a good synchronization between subjective off and gait off (see patients no. 24–33, Table 2). For example, patient no. 28 (Figure 4(a)) felt “good” between 1000 and 1200 and between 800 and 900. These symptoms correlated well with the increase in cadence and acceleration (activity-related changes), suggesting high physical activity. In contrast, the patient felt “bad” between 1400 and 1600 and at 1800, and “not good” between 1700 and 2100–2200. Coinciding with the subjective off, the cadence and acceleration decreased simultaneously at 1500, indicative of bradykinetic walking. Patient no. 27 (Figure 4(b)) felt “not good” or “bad” due to the frequent occurrence of the freezing of gait between 0600 and 1200. The PGR showed changes that coincided with the freezing of gait: an increase of cadence and decrease of acceleration, at 0800 and 900.

In the other 11 (52.4%) patients, the gait parameters did not correlate with the subjective complaints. In three patients (patients no. 34–36), the PGR findings of gait off were not synchronized with the subjective off findings. For example, patient no. 35 (Figure 4(c)) felt “bad” between 1200 and 1500, whereas no changes were noted in cadence. In the other eight patients (no. 37–44), the off time was different between subjective and PGR recordings. Patient no. 38 (Figure 4(d)) felt “not good” at 1500. However, the gait cadence and acceleration were within the normal ranges at 1500. At 1800, the patient walked with a marked bradykinetic stepping. No significant difference was observed in UPDRS-III between patients with matched and unmatched symptoms—PGR.

 Taken together, desynchronization was observed between the gait fluctuation and subjective fluctuation in 30 of the 44 (68.2%) patients whose 2- hours profiles were determined by PGR.

## 4. Discussion

### 4.1. Quantification of Motor Fluctuation in Patients with Gait Disorders

The aim of the present study was to examine the relationship between complaints of PD patients and motor fluctuation. Definition of such relationship requires recording and quantification of motor fluctuation. We focused on the parkinsonian gait disorder which can be quantified easily by extracting data from the gait cadence and acceleration. These two parameters have the following different physiological characteristics.

Previous studies showed that (1) any increase in the cadence beyond the mean + 1SD is associated with a marked short stepping or freezing and (2) any decrease in cadence below the mean − 1SD is associated with a walking pattern characterized by a marked bradykinetic stepping. Thus, the off period reflects the time when the cadence deviates from the range of mean ± 1SD of the control subjects. In contrast, it is difficult to determine the “on or off” based on the value of gait acceleration alone. This is based on the extremely wide range of the acceleration in daily life walking (Figure 3 of [9]). Furthermore, the absolute value of the acceleration could be variable depending on the measurement conditions [12].

These features of the two parameters allowed the definition of criteria for identifying gait bradykinetic fluctuation. We first defined candidates of *gait off* and *gait good* based on changes in the cadence. Second, we tested this validity by examining whether corresponding changes were observed in the gait acceleration.

### 4.2. Comparison between Cadence Fluctuation and Acceleration Fluctuation

Cadence fluctuations beyond the intersubject mean ± 1SD of the normal control (normal range) were presumed to represent motor fluctuation (*probably gait off*). We further tested the validity of these fluctuations based on the acceleration change. In the first stage, we excluded activity-related increases in the cadence. Physical activity was associated with increased cadence and acceleration, due to fast stepping during walking and augmentation of floor reaction force [14].

In the second stage of the study, we matched the changes in cadence to those in acceleration. During the off period, the amplitude of the acceleration should decrease, since the ability to disturbances in force production. In the present study, the cut-off level was defined as the mean − 2SD of the control subjects. We demonstrated in our previous studies that acceleration below the mean − 2SD was associated with severe disturbances in the production of floor reaction forces (Figure 3 of [9]), indicating that this range reflects the off-related changes. Based on the above criteria, we examined whether deviations of the cadence beyond the normal range were associated with a simultaneous decrease in the acceleration less than the cut-off level. No such relationship was identified in 4 of 41 patients. In the other 37 (90.2%) patients, however, a total harmony was observed between the cadence and the acceleration changes. We labeled these patients to have *definite gait off*.

On the other hand, cadence within the normal range throughout the day suggested the absence of motor fluctuation during the recording. In patients with *probable gait good*, we further excluded patients with large variability in accelerations. For this purpose, we calculated the coefficient variance (CV) of acceleration in control subjects. We defined the cut-off level as 0.24, corresponding to the intersubject mean + 1SD of the control subjects. Any change in acceleration beyond this cut-off level should mean unlikely fluctuation in the control subjects. Based on these definitions, 6 of the 13 patients with *probable constant* showed fluctuation that hardly occurred in the control subjects. The other 7 patients (53.8%) with CV values below the cut-off level were assessed as *definite gait good*.

### 4.3. Desynchronization between Gait Fluctuation and Subjective Fluctuation

Based on the changes in cadence and acceleration (gait fluctuation), we then assessed the agreement between gait fluctuation and subjective fluctuation. Interestingly, no such agreement was observed in 30 of the 44 patients (68.2%). These cases were classified into the following two patterns. One major pattern was “no consciousness.” Definite gait fluctuation occurred in 19/23 (82.7%) patients who subjectively felt no fluctuation. On the other hand, gait fluctuation was not recorded or did not synchronize with the subjective symptoms in 11/21 (52.4%) patients.

What is the reason for the lack of synchronization? One reason for the discrepancy could be that the patient concerns or priorities varied from one patient to another. In some patients, the target of attention might be other types of movement disorders. For example, some patients might feel good when they could easily manipulate their hands. Other patients might feel subjectively good upon the improvement of hypokinesia (difficulty in initiation of movements). In such patients, they would estimate to be on time when they easily initiated their movements without hesitation. Moreover, other patients might weigh mood disorders such as apathy, rather than movement disorders. Thus, the present finding of desynchronization between subjective off and gait off could represent the inherent features of PD itself. Another alternative explanation is that some PD patients did not notice motor fluctuation due to deficits in attention to motor deficits [5]. In such a condition, fluctuation in the gait parameters would have occurred even though the patients felt good throughout the day. Further studies are needed to perform an advanced statistica analysis, for example, a logistic regression analysis to adjust for these confounding factors. The limitation of the present methodology also should be noticed since our device will only be useful in individuals who have specific gait disorders.

## 5. Conclusion

The present results showed that subjective assessment does not necessarily match the findings of quantitative objective assessment in PD patients with gait disorders. The results highlight the importance of true assessment of the patients' complaints to identify the wearing off. For this purpose, an objective long-term monitoring system, including PGR, would be helpful.

## Figures and Tables

**Table 1:** Clinical features of patients with Parkinson's disease.

Case no.	Age (years)	Gender	Duration (years)	M H-Y	UPDRS Part III				Medication
					Total score	Gait	Postural stability	Bradykinesia
Subjective good									

1	73	M	3.6	3	12	1	2	2	L-dopa 300 mg, Ama 200 mg
2	73	M	3.6	3	12	1	2	2	L-dopa 300 mg, Ama 200 mg
3	79	M	1.3	3	44	2	2	2	L-dopa 300 mg
4	70	F	2.8	3	11	1	2	1	L-dopa 300 mg, Pra 1.5 mg
5	55	M	2.5	2	11	0	0	0	Rop 4 mg, Ama 200 mg
6	70	F	3.6	2.5	9	1	1	1	L-dopa 300 mg
7	75	M	3.0	3	23	1	2	2	None
8	64	F	2.6	3	16	2	1	1	Rop 2 mg, Ama 200 mg
9	73	F	7.7	1.5	10	0	0	0	L-dopa 300 mg, Pra 3 mg, Ama 100 mg
10	83	M	3.7	2	12	2	0	1	Ama 100 mg
11	63	F	0.5	2	23	1	0	1	None
12	76	M	3.1	2	16	0	0	0	L-dopa 300 mg
13	79	M	3.5	3	34	1	2	2	L-dopa 300 mg
14	79	M	3.5	2.5	29	1	1	2	L-dopa 200 mg, Tri 4 mg
15	78	M	8.3	2	11	0	0	0	None
16	79	M	3.8	3	23	1	2	1	L-dopa 300 mg, Pra 1.5 mg
17	78	M	2.9	2.5	14	1	1	1	L-dopa 300 mg
18	75	F	2.4	3	35	2	2	2	L-dopa 300 mg, Pra 1.0 mg
19	77	M	17.6	3	30	2	1	3	Pra 2.0 mg, Ama 100 mg
20	75	M	11.7	3	26	1	2	2	L-dopa 300 mg
21	64	M	1.8	1.5	10	0	1	1	L-dopa 300 mg, Pra 1.5 mg
22	63	F	1.3	2	21	0	0	1	None
23	63	F	1.4	2	19	0	0	1	Pra 2 mg

Subjective not good									

24	76	M	17.3	3	36	2	2	3	Ama 100 mg
25	67	M	3.3	2	30	1	1	1	L-dopa 300 mg, Ama 200 mg
26	71	M	3.4	3	29	1	1	1	L-dopa 300 mg, Rop 4 mg
27	69	M	1.0	3	17	1	2	2	None
28	59	M	9.0	2	15	0	0	1	L-dopa 300 mg, Pra 3 mg, Sel 5 mg, Ama 200 mg
29	77	M	2.8	2.5	21	1	1	1	L-dopa 400 mg
30	82	M	3.9	4	31	2	1	2	L-dopa 300 mg, Rop 3 mg
31	64	M	1.5	1.5	8	0	0	1	L-dopa 300 mg
32	79	M	7.7	3	21	2	1	1	L-dopa 400 mg, Per 1.25 mg, Sel 5 mg
33	78	F	2.0	3	31	1	2	3	None
34	81	F	8.2	3	17	1	2	2	L-dopa 300 mg, Rop 2 mg
35	65	F	3.3	2.5	19	1	2	2	Pra 1 mg, Ama 200 mg
36	77	F	9.6	2.5	23	1	1	1	L-dopa 300 mg, Rop 2 mg
37	56	F	2.3	3	18	1	2	2	L-dopa 300 mg, Rop 9 mg, Ama 200 mg
38	73	F	0.5	3	11	0	2	1	None
39	74	F	2.3	3	35	2	2	2	L-dopa 300 mg
40	70	F	2.8	3	14	1	2	1	L-dopa 300 mg
41	66	F	2.7	2.5	15	1	1	1	L-dopa 200 mg, Rop 6 mg
42	72	M	8.3	3	24	1	1	1	L-dopa 400 mg, Rop 5 mg
43	65	M	1.7	1.5	10	1	0	0	Tri 4 mg
44	64	F	12.8	3.5	21	1	2	1	L-dopa 500 mg, Pra 4 mg, Ent 500 mg

M H-Y: modified Hoehn and Yahr stage, UPDRS: unified Parkinson's disease rating scale, gait: “gait” score, postural stability: “postural stability” score, bradykinesia: “body bradykinesia and hypokinesia” score, Ama: amantadine, Pra: pramipexole, Rop: ropinirole, Tri: trihexyphenidyl, Sel: selegiline, Ent: entacapone, subjective good: patients who did not notice wearing off, and subjective wearing off: patients who noticed subjectively wearing off.

**Table 2:** Comparison between gait off and subjective off.

Case no.	Gait off time	Subjective off time	Synchronization
Subjective good			

1	Good	Good	Both good
2	Good	Good	Both good
3	Good	Good	Both good
4	Good	Good	Both good
5	0600	Good	No
6	0800	Good	No
7	1600	Good	No
8	1900	Good	No
9	0400, 0500	Good	No
10	0700, 1600	Good	No
11	0200, 1900	Good	No
12	2200, 2300	Good	No
13	1000, 1300, 1800	Good	No
14	1000, 1100, 1600	Good	No
15	0100, 2000, 2100	Good	No
16	1400, 1600, 1800, 2200	Good	No
17	0600, 1700, 1900, 2000	Good	No
18	0800, 0900, 1800, 2100	Good	No
19	0500, 0700, 0800, 2000	Good	No
20	0100, 0800, 1700, 1800, 2100	Good	No
21	0800, 0900, 1800, 2100, 2400	Good	No
22	0600, 0800, 0900, 1200, 1300, 1800, 1900, 2000, 2100	Good	No
23	0500, 0600, 0700, 0800, 1200, 1800, 1900, 2000, 2100, 2200	Good	No

Subjective wearing off			

24	0800	0800, 2100	Both off at 0800
25	1300	1200, 1300, 1600	Both off at 1300
26	1700, 1800	1600, 1700	Both off at 1700
27	0800, 0900, 1400	0600, 0700, 0800, 0900, 1000, 1100, 1200	Both off at 0800, 0900
28	0600, 0700, 01500	1400, 1500, 1600, 1800	Both off at 1500
29	1500, 1800, 1900	0600, 0700, 1300, 1400, 1800, 1900, 2000, 2100, 2200	Both off at 1800, 1900
30	0200, 0800, 1200, 1300	0800	Both off at 0800
31	0800, 0900, 1500, 1700, 1900, 2000, 2400	0900, 1000, 1100, 1300	Both off at 0900
32	0100, 0300, 0800, 1700, 1900, 2000	0800	Both off at 0800
33	0700, 0800, 0900, 1100, 1200, 1700, 1800, 1900, 2000, 2100	0800, 1200, 1400, 1500, 1800, 1900, 2000, 2100, 2200	Both off at 1200, 1800, 2000, 2100
34	Good	0900, 1000, 1100, 1700, 1800, 1900	No
35	Good	1200, 1300, 1400, 1500	No
36	Good	1600	No
37	1500	1000, 1100, 1800, 1900	No
38	1800	15	No
39	2000	13, 14, 15	No
40	0100, 0700, 0800	11, 12, 13, 17, 18, 19, 23	No
41	0800, 0900, 1400	16, 17, 18, 19, 20	No
42	0100, 0400, 1300, 2300	5, 7, 15, 16, 17, 19, 20, 21	No
43	0400, 0600, 0700, 1500	18	No
44	0300, 0600, 0700, 1600	12, 13	No

Gait off time: the time when the gait off was recorded. Subjective off time: the time when the subjective gait off was noticed. Subjective good: patients who did not notice wearing off. Subjective wearing off: patients who noticed subjective wearing off.
